# Maternal polymorphic loci of rs1979277 *serine hydroxymethyl transferase* and rs1805087 *5-methylenetetrahydrofolate* are correlated with the development of fetal growth restriction: A case-control study

**DOI:** 10.18502/ijrm.v19i12.10057

**Published:** 2022-01-12

**Authors:** Olesya Efremova, Irina Ponomarenko, Mikhail Churnosov

**Affiliations:** Department of Biomedical Disciplines, Belgorod State University, Belgorod, Russia.

**Keywords:** Polymorphism, Associations, Fetal growth restriction, Folic acid.

## Abstract

**Background:**

Key reactions in folate-mediated single-carbon metabolism are regulated by folate cycle enzymes. Violations of the folate cycle may be associated with the occurrence of fetal growth restriction (FGR) in pregnant women.

**Objective:**

To study the relationship between polymorphisms of folate cycle genes in the mother with the development of FGR.

**Materials and Methods:**

In this case-control study, 122 pregnant women with FGR and 243 pregnant women with normal newborn weight were enrolled. The polymorphic loci of folate cycle genes including rs1805087 5-methylenetetrahydrofolate (*MTR*) and rs1979277 serine hydroxymethyl transferase (*SHMT1*) were examined. The study of polymorphisms was carried out through the TaqMan probe detection method using polymerase chain reaction. Logistic regression was used to analyze the associations of the polymorphisms.

**Results:**

It was established that the T allele rs1979277 of the *SHMT1* gene was correlated with the development of FGR within the framework of the allelic (OR = 1.67, 95% CI 1.20-2.33, p ${}_{\text{perm}}$ < 0.01), additive (OR = 1.69, 95% CI 1.20-2.37, p ${}_{\text{perm}}$ < 0.01), dominant (OR = 1.81, 95% CI 1.15-2.87, p ${}_{\text{perm}}$ = 0.01) and recessive (OR = 2.34, 95% CI 1.15-4.73, p ${}_{\text{perm}}$ = 0.01) models. The association of the G rs1805087 allele of the *MTR* gene with the occurrence of FGR was also identified following the recessive model (OR = 3.01, 95% CI 1.05-8.68, p ${}_{\text{perm}}$ = 0.04).

**Conclusion:**

Our results indicated that maternal polymorphic loci rs1979277 *SHMT1* and rs1805087 *MTR* may be associated with the development of FGR.

## 1. Introduction 

Problems in folic acid metabolism can cause a range of consequences that complicate the course of pregnancy (1-3). Folic acid is known to be involved in the formation of the vascular bed. Changes in angiogenesis can cause placental dysfunction, which is associated with the pathogenesis of fetoplacental insufficiency and can lead to fetal growth restriction (FGR) (4, 5). FGR is a condition where the rate of fetal growth is lower than expected for the gender and race of the fetus (6, 7). Over the past few decades, the frequency of FGR and placental insufficiency has increased in many countries (8, 9).

Folic acid metabolism is carried out through a complex cascade process, accompanied by genetically determined enzymatic reactions, and occurs in most organs, including the placenta (2, 4). The serine hydroxymethyl transferase gene (*SHMT1*) encodes a pyridoxal phosphate-dependent enzyme that catalyzes the interconversion of serine and glycine, and enables the folate-dependent single-carbon metabolism necessary for the synthesis of purines and thymidylate, as well as for the conversion of homocysteine to methionine. Methionine is subsequently adenylated to S-adenosylmethionine, a cofactor that methylates deoxyribonuclease, ribonuclease, proteins, and many metabolites (10, 11).

Folate absorption occurs in specialized and multinucleated placental cells in the presence of the enzyme encoded by 5-methylenetetrahydrofolate (*MTR)*. Methionine synthase is expressed in the villous syncytiotrophoblast, and 5,10-methylenetetrahydrofolate reductase is expressed in the extravillous trophoblast. It has been shown that the *MTR*-encoded enzyme in the villus trophoblast is involved in the metabolism of homocysteine using folate. The gene methionine synthase reductase (*MTRR*) encodes the cytoplasmic enzyme methionine synthase reductase, one of the functions of which is to reverse the conversion of homocysteine to methionine (10, 11).

Thus, metabolic enzymes such as 5,10-methylenetetrahydrofolate reductase and serine hydroxymethyl transferase in the mother's body play an important role in monocarbon folate metabolism and normal fetal development. Understanding the role of the *SHMT1* gene polymorphisms in the process of intrauterine growth restriction is important for the development of effective methods for the diagnosis and prevention of this pregnancy complication.

This study aimed to evaluate the association between folate cycle gene polymorphisms in the maternal body with the development of FGR.

## 2. Materials and Methods

### Design and participants

In this case-control study, we recruited 365 pregnant women in their third trimester, from whom anamnestic data were collected, and general clinical and biochemical parameters were studied. Given the available data about the allele frequencies of the studied folate cycle gene polymorphisms in the European population (data of the 1000 Genomes Project), we calculated that sample size of 365 should be sufficient to ensure the statistical power of 0.80 at α = 0.05 significance level. This research was conducted at the Regional Perinatal Center of the city of Belgorod in the Russian Federation, from June 2014 to December 2018. Participants included 122 pregnant women with FGR (defined as fetal weight of 10 or more percentiles below the standard) as the case group, and 243 pregnant women with normal birth weight as a control group. The diagnosis of FGR was based on clinical data, parameters of growth, weight after the birth, and ultrasound fetometry (TOSHIBA XARIO SSA-660A, manufacturer Toshiba (Canon), Japan) (4, 7). The sample for the genetic testing was taken only from the mother. The inclusion criteria were as follows: рatients in the third trimester of pregnancy; with spontaneous singleton pregnancy; and FGR. The control group consisted of pregnant women with a normally developed fetus. Exclusion criteria included: multiple pregnancies; treatment with insulin therapy for gestational diabetes mellitus; diagnosis in the mother of human immunodeficiency virus, viral hepatitis, or severe uncompensated extragenital diseases; diagnosis in the fetus of hemolytic disease, anomaly of fetal development, antiphospholipid syndrome, or congenital thrombophilia; or circulatory disorders in the mother-placenta-fetus interface.

### Genetic measurements

DNA was extracted from the venous blood of the pregnant women using the phenol-chloroform method and was then checked for quality as described previously (12, 13). Five single nucleotide polymorphisms (SNPs) were selected for the analysis, based on having significant regulatory potential (14, 15): *MTR* (rs1805087)*,* *MTRR *(rs1801394)*,* *SHMT1 *(rs1979277) and *TYMS *(rs699517, rs2790). The study was carried out through polymerase chain reaction using appropriate oligonucleotide primers and probes. Then the polymorphisms were analyzed using the detection method of TaqMan probes (real-time polymerase chain reaction).

### Ethical considerations

This study was approved by the Ethical Committee of the Medical Institute of Belgorod State University (reference number: 54). The study details were explained to the women before they participated in the study, and informed consent was obtained from all.

### Statistical analysis

Statistical analysis of the biomedical and clinical characteristics of the studied groups was carried out using the STATISTICA 7,0 for Windows 10.0 software package. Differences in the studied traits between the compared independent groups (pregnant women with FGR and control) were evaluated using the Mann-Whitney test. Logistic regression was used to assess the associations between the clinical and clinical-anamnestic risk factors, and the development of FGR. The odds ratios (OR) and their 95% confidence intervals (95% CI) were calculated (16).

Logistic regression was also used to assess associations of the SNPs with FGR assuming additive, recessive, and dominant genetic models (17). Statistical calculations were performed using the gPLINK v2.050 software (http://zzz.bwh.harvard.edu/plink/). To correct for multiple comparisons, a permutation test was used (18).

## 3. Results 

The case group (n = 122) and the control group (n = 243) did not differ by age or height of the pregnant women (Table I). The father's age also did not differ by groups: case group - 27.3 ± 7.7 yr; control group - 26.9 ± 6.2 yr (p = 0.69).

The findings showed that the women with FGR had a significantly lower weight before pregnancy than the control group (p = 0.01). The body mass index of the case group was also significantly lower than the control group (p < 0.01). The mean weight of the case group newborns was 2147.26 ± 621.15 g and in the control group was 3463.26 ± 438.26 g (p < 0.01). The growth of newborns in the case group was 40.27 ± 2.41 cm and in the control was 54.51 ± 2.26 cm (p < 0.01) (Table I).

For all of the studied SNPs, both in the case group and the control group, the frequencies of minor alleles were higher than 5%. For all the examined loci in both groups, the analysis of the observed distribution of genotypes did not reveal deviations from the expected distribution following the Hardy-Weinberg equilibrium (Table II).

An analysis of the association between folate cycle gene polymorphic loci alleles and the development of FGR (Table III) showed that the T rs1979277 allele of the *SHMT1* gene was significantly associated with the development of FGR (OR = 1.67, 95% CI 1.20 - 2.33, p < 0.01, p ${}_{\text{perm}}$ < 0.01, N ${}_{\text{perm}}$ = 6342).

It was found that the T allele rs1979277 of the *SHMT1* gene was associated with the development of FGR within the additive (OR = 1.69, 95% CI 1.20-2.37, p < 0.01, p ${}_{\text{perm}}$ < 0.01, N ${}_{\text{perm}}$ = 8235), dominant (OR = 1.81, 95% CI 1.15-2.87, p = 0.01, p ${}_{\text{perm}}$ = 0.01, N ${}_{\text{perm}}$ = 1706), and recessive (OR = 2.34, 95% CI 1.15-4.73, p = 0.02, p ${}_{\text{perm}}$ = 0.01, N ${}_{\text{perm}}$ = 1352) models (Table IV). The association of the G rs1805087 allele of the *MTR* gene with the formation of FGR was also identified in accordance with the recessive model (OR = 3.01, 95% CI 1.05-8.68, p = 0.04, p ${}_{\text{perm}}$ = 0.04, N ${}_{\text{perm}}$ = 506).

**Table 1:** The main biomedical and medical history indicators in the study groups


**Parameters**	**FGR (n = 122)**	**Control (n = 243)**	**p-value**
**Height of women before pregnancy (m)**	1.68 ± 0.53	1.63 ± 0.68	0.247
**Age of pregnant women (yr)**	25.48 ± 5.34	26.47 ± 5.63	0.547
**Weight before pregnancy (kg)**	64.43 ± 10.59	67.22 ± 11.54	0.008
**BMI before pregnancy (kg/m^2^)**	22.13 ± 4.20	24.25 ± 3.56	0.003
**Weight of the newborn (g)**	2147.26 ± 621.15	3463.26 ± 438.26	0.001
**Newborn growth (cm)**	40.27 ± 2.41	54.51 ± 2.26	0.004
Data are presented as Mean ± SD. Mann-Whitney nonparametric test was used. BMI: Body mass index, FGR: Fetal growth restriction			

**Table 2:** Distribution of the five polymorphic loci of folate cycle genes in both groups


**Parameters**	**FGR (n = 122)**					**Control (n = 243)**				
**CHR**	1	5	17	18	18	1	5	17	18	18
**SNP**	rs1805087	rs1801394	rs1979277	rs699517	rs2790	rs1805087	rs1801394	rs1979277	rs699517	rs2790
**Gene**	*MTR*	*MTRR*	*SHMT1*	*TYMS*	*TYMS*	*MTR*	*MTRR*	*SHMT1*	*TYMS*	*TYMS*
**Minor allele**	G	A	T	T	G	G	A	T	T	G
**Frequent allele**	A	G	C	C	A	A	G	C	C	A
**Minor allele frequency**	0.244	0.492	0.396	0.292	0.210	0.225	0.430	0.281	0.294	0.167
**Number of chromo-somes studied**	238	238	230	236	238	454	486	462	470	484
Genotype distribution*	9/40/70	27/63/29	18/55/42	9/51/58	6/38/75	6/90/131	44/121/78	17/96/118	18/102/115	7/67/168
**H ${}_{0}$ **	0.336	0.529	0.478	0.432	0.319	0.396	0.498	0.416	0.434	0.277
**H ${}_{е}$ **	0.369	0.500	0.478	0.414	0.332	0.348	0.490	0.404	0.415	0.279
**P ${}_{\text{HWE}}$ **	0.325	0.585	1.000	0.824	0.781	0.055	0.896	0.747	0.532	0.821
*Count of homozygotes for the minor allele/heterozygotes/homozygotes for the frequent allele. Logistic regression model was used. CHR: Chromosome, SNP: Single nucleotide polymorphism, FGR: Fetal growth restriction, H ${}_{0}$ : Observed heterozygosity, H ${}_{е}$ : Expected heterozygosity, *MTR*: 5,10-methylenetetrahydrofolate reductase, *MTRR*: Methionine synthase reductase, *SHMT1*: Serine hydroxymethyl transferase 1, *TYMS*: Thymidylate synthetase. P ${}_{\text{HWE}}$ : Significance level for correspondence to the Hardy-Weinberg equilibrium (Chi-square test was used)										

**Table 3:** Associations of five polymorphic loci of folate cycle gene alleles with FGR


**CHR**	**SNP**	**Gene**	**Minor allele**	**Minor allele frequency**		**OR (95% CI)**	**p-value**
		**FGR**	**Control**	
**1**	rs1805087	*MTR*	G	0.244	0.225	1.11 (0.77-1.61)	0.57
**5**	rs1801394	*MTRR*	A	0.492	0.430	1.28 (0.94-1.75)	0.12
**17**	rs1979277	*SHMT1*	T	0.396	0.281	1.67 (1.20-2.33)	< 0.01
**18**	rs699517	*TYMS*	T	0.292	0.294	0.99 (0.70-1.40)	0.97
**18**	rs2790	*TYMS*	G	0.210	0.167	1.32 (0.89-1.96)	0.16
CHR: Chromosome, SNP: Single nucleotide polymorphism, FGR: Fetal growth restriction, OR: Odds ratio, CI: Confidence interval, *MTR*: 5,10-methylenetetrahydrofolate reductase, *MTRR*: Methionine synthase reductase, *SHMT1*: Serine hydroxymethyl transferase 1, *TYMS*: Thymidylate synthetase. Logistic regression was used. Statistically significant results were selected taking into account the permutation test (1000 permutations were performed)							

**Table 4:** Results of logistic regression analysis of the associations of five SNPs of folate cycle genes with the development of FGR in scope of additive, dominant and recessive models


**CHR**	**SNP**	**Gene**	**N**	**Additive model**		**Dominant model**		** Recessive model**	
		**OR (95% CI)**	**p-value**	**OR (95% CI)**	**p-value**	**OR (95% CI)**	**p-value**
**1**	rs1805087	*MTR*	346	1.12 (0.76-1.64)	0.56	0.95 (0.61-1.50)	0.84	3.01 (1.05-8.68)	0.04
**5**	rs1801394	*MTRR*	362	1.29 (0.94-1.77)	0.11	1.47 (0.89-2.41)	0.13	1.33 (0.77-2.28)	0.30
**17**	rs1979277	*SHMT1*	346	1.69 (1.20-2.37)	< 0.01	1.81 (1.15-2.87)	0.01	2.34 (1.15-4.73)	0.02
**18**	rs699517	*TYMS*	353	0.99 (0.70-1.41)	0.97	0.99 (0.64-1.54)	0.97	0.99 (0.43-2.29)	0.10
**18**	rs2790	*TYMS*	361	1.31 (0.89-1.94)	0.17	1.33 (0.84-2.11)	0.22	1.78 (0.58-5.43)	0.31
CHR: Chromosome, SNP: Single nucleotide polymorphism, FGR: Fetal growth restriction, OR: Odds ratio, CI: Confidence interval, *MTR*: 5,10-methylenetetrahydrofolate reductase, *MTRR*: Methionine synthase reductase, *SHMT1*: Serine hydroxymethyl transferase 1, *TYMS*: Thymidylate synthetase. Logistic regression was used. Statistically significant results were selected taking into account the permutation test (1000 permutations were performed)									

## 4. Discussion 

In our study, it was found that polymorphic loci rs1979277 of the *SHMT1* gene and rs1805087 of the *MTR* gene in the mother were associated with the development of FGR. It was identified that the alleles T rs1979277 of the *SHMT1* gene and G rs1805087 of the *MTR* gene were associated with an increased risk of FGR development (OR = 1.67-2.34 and OR = 3.01 respectively).

The results obtained are in accordance with the literature on the medico-biological effects of the studied genes. Epidemiological and experimental data consistently point to an association between folate deficiency in the first trimester of pregnancy, and poor fetal development and health of the offspring (19). Genetic disorders affecting folic acid metabolism have been found in patients with lung cancer (20). Some loci and genes that are associated with folic acid levels have been found using genome-wide associative studies, such as rs1801133 in *MTR* and rs1979277 in *SHMT1* (21). The role of folic acid in reproductive activity has been shown. A number of authors have demonstrated that folic acid improves sperm quality and reduces the negative effect of high doses of drugs on sperm (22, 23). In many countries, to prevent the development of birth defects in the fetus, pregnant women are prescribed folic acid (4, 24). The role of the *MTR* A2756G polymorphism in the development of idiopathic male infertility has been demonstrated (25). *MTR* rs1805087 has been shown to have a statistically significant effect on the methylation levels of DNA methyltransferase 1, which is responsible for maintaining DNA methylation patterns during cell division (26).

A number of studies indicate that mitochondrial *SHMT*-derived monocarbon units are necessary for mediated folate of single-carbon metabolism in the cytoplasm (27). The relationship of *SHMT1* gene polymorphisms with pregnancy and fetal development was demonstrated by Fekete et al. (28). Thus, in one case-control study, the relationship between *SHMT1*, dietary folic acid intake, preterm labor, and FGR was studied. It was shown that Caucasian carriers of *SHMT1* T had an increased risk of spontaneous preterm delivery and development of FGR (2). Studies have also been carried out to examine the associations of *SHMT1* with the development of acute lymphoblastic leukemia, tumors, neural tube defects, and sclerotic changes (29-32).

It has been shown that a polymorphic variant of the *SHMT1 *gene affects the occurrence of intrauterine malformations. One study found that the rs1979277 A allele reduced the cytoplasmic activity of *SHMT* and had a higher frequency in the control vs. cases with non-syndromic cleft lip; the authors therefore suggested that a low enzyme activity may increase the cytoplasmic concentration of folates (33). *SHMT1* provides the single-carbon units necessary for embryogenesis, and defects in the production of carbon alone lead to certain pathological conditions during pregnancy. Using intrauterine, maternal, and paternal groups and both triad and family approaches, it has been shown that the interaction between maternal and paternal *SHMT1* C1420T predisposes the fetus to neural tube defects (34).

## 5. Conclusion 

As a result of the study, a possible association of maternal polymorphic loci rs1979277 *SHMT1* and rs1805087 *MTR* with FGR was established.

##  Conflict of Interest

The authors declare that they have no competing interest.
